# Development and first assessment of a questionnaire for health care utilization and costs for cardiac patients

**DOI:** 10.1186/1472-6963-8-187

**Published:** 2008-09-19

**Authors:** Bernd Schweikert, Harry Hahmann, Reiner Leidl

**Affiliations:** 1 Institute of Health Economics and Health Care Management, Helmholtz Zentrum München, German Research Center for Environmental Health, P.O. Box 1129, D-85758 Neuherberg, Germany; 2 Institute of Health Economics and Management, Ludwig-Maximilians-University Munich, Munich, Germany; 3 Klinik Schwabenland, Isny-Neutrauchburg, Germany

## Abstract

**Background:**

The valid and reliable measurement of health service utilization, productivity losses and consequently total disease-related costs is a prerequisite for health services research and for health economic analysis. Although administrative data sources are usually considered to be the most accurate, their use is limited as some components of utilization are not systematically captured and, especially in decentralized health care systems, no single source exists for comprehensive utilization and cost data. The aim of this study was to develop and test a questionnaire for the measurement of disease-related costs for patients after an acute cardiac event (ACE).

**Methods:**

To design the questionnaire, the literature was searched for contributions to the assessment of utilization of health care resources by patient-administered questionnaires. Based on these findings, we developed a retrospective questionnaire appropriate for the measurement of disease-related costs over a period of 3 months in ACE patients. Items were generated by reviewing existing guidelines and by interviewing medical specialists and patients. In this study, the questionnaire was tested on 106 patients, aging 35–65 who were admitted for rehabilitation after ACE. It was compared with prospectively measured data; selected items were compared with administrative data from sickness funds.

**Results:**

The questionnaire was accepted well (response rate = 88%), and respondents completed the questionnaire in an average time of 27 minutes. Concordance between retrospective and prospective data showed an intraclass correlation (ICC) ranging between 0.57 (cost of medical intake) and 0.9 (hospital days) with the other main items (physician visits, days off work, medication) clustering around 0.7. Comparison between self-reported and administrative data for days off work and hospitalized days were possible for n = 48. Respective ICCs ranged between 0.92 and 0.94, although differences in mean levels were observed.

**Conclusion:**

The questionnaire was accepted favorably and correlated well with alternative measurement approaches. This first assessment showed promising characteristics of this questionnaire in different aspects of validity for patients with ACE. However, additional research and more extensive tests in other patient groups would be worthwhile.

## Background

The valid and reliable measurement of health service utilization, productivity losses and consequently total disease-related costs is a prerequisite for health economic analysis. While health effects can be measured by numerous generic and disease-specific instruments [1-3], much less work has been done regarding standardized methods to measure health care utilization and disease-specific costs.

Particularly when an economic evaluation is performed prospectively alongside a clinical trial, cost data may be collected by various methods, such as clinical records, insurance data, and data from health care providers. Patients may also provide data directly, by face to face interviews, retrospective cost questionnaires, and prospective cost diaries. Overall, health economic studies commonly use cost data from administrative data sources and routine databases from hospitals, health insurances, or health care providers. They are usually considered to be the most accurate method of cost measurement [4,5]. However, the use of administrative data has some significant disadvantages. First, in economic evaluations adopting a societal perspective which includes all medical and non-medical costs and potential savings related to the disease studied is broadly recommended [6,7]. Administrative data, however, will typically miss some cost components that should be considered. These include expenditures for over the counter drugs, travel expenses, or costs resulting from paid or unpaid informal care. These cost items are also essential if one is interested in the economic impact on patients by different types of treatment. Second, in decentralized health care systems, including many social security systems and market-oriented health systems, no single source exists for comprehensive cost data. When study patients are insured by multiple payers or receive their medical care at multiple sites, investigators must contact numerous third party payers and service providers in order to collect comprehensive cost data. In this case, the use of administrative data may not be feasible and may be inefficient even for a small number of patients. Thus in practice, investigators may rely to some degree on patient-reported data. However, this may lead to other problems typical of survey studies, such as non-response and missing data. Face to face interviews limit these problems but may be too costly for large numbers of patients [8]. Diaries are considered to be feasible and valid, and are advantageous in that they do not rely on instantaneous recall [9]. They allow information to be collected prospectively, which reduces the likelihood of recall error. However, once the diary is sent, it is impossible to determine whether the information was filled in promptly or after receiving reminder letters, which might be weeks later. While cost diaries are well accepted over the short term, significant problems have been reported over longer time periods, with drop-out rates of up to 50% of the study population over one year [8,10,11]. Similar problems arise with diaries for clinical trials and related health economic studies, where follow-up periods of 6–12 months are typical. In this case, a retrospective approach may be attractive. However, available retrospective instruments are rarely validated [9,12]. To our knowledge, there is no tested instrument available that was designed to comprehensively measure the full range of direct and indirect medical and non-medical costs alongside a clinical trial in cardiac patients.

The aim of this study was to develop and test a patient-administered instrument for a clinical trial, which would allow comprehensive cost measurements related to cardiac disease over a longer time period without exceeding normal recall periods.

Thus, the objective of this article is (1) to introduce the instrument and its development, (2) to assess its acceptance and feasibility, and aspects of its validity in a clinical trial setting, (3) to identify the cost structure and the overall level of disease-related costs in patients after an acute cardiac event.

## Methods

### Questionnaire development

The cost questionnaire was designed as a booklet with instructions that allow the patients to complete it independently; it was based on the format and wording of cost diaries developed and introduced in studies of patients with low back pain [13] and inflammatory bowel disease [14].

An enclosed letter explained the study's aim and design and a telephone number was provided in case of questions. Patients were instructed at the beginning of the questionnaire to complete it alone. The cost items were adapted to the specific utilization pattern of cardiac patients after an acute event. These were based on published treatment guidelines and interviews with cardiac patients, cardiologists in private practice, and a physician at the rehabilitation clinic.

"Filter questions" were used for each cost item to clarify and to the improve user-friendliness of the questionnaire items. These asked whether any of the subsequent disease-related costs were incurred during the respective time frame. Only if the subject answered "yes" were they required to complete a subsequent table, in which frequency and/or cost of utilization were to be specified. In addition, the use of filter questions allowed "no" answers to be distinguished from missing answers, which is an important distinction when calculating mean costs.

A crucial issue in the development of the questionnaire was to determine the appropriate time frame for the single cost items based on literature regarding cognitive theory, empirical results in survey research, and a recent review in the field of health care [15]. The following factors were identified and considered when determining the time frame and the framing of questions in order to balance underreporting due to recall error and potential overreporting due to so called "telescoping" effects. These are errors in reporting incurred by the temporal displacement of an event, which typically leads respondents to overreport utilization frequencies [16]:

• There is no single ideal time frame for the recall period of a cost questionnaire. The appropriate recall time span for different utilization variables differs with respect to the nature of cost items [17-21].

• The less regularly an event occurs and the lower its subjective impact is, the less likely it is to be recalled exactly over the longer time frame [22].

• Respondents estimate, rather than count, relatively frequent events. A longer time frame does not improve data quality [11,23].

• Techniques of "bounded recall" should be used if possible [24]. This means, that the time horizon is explicitly defined by exact dates and, for repeated measurements, the preceding answers and already recalled events should be shown.

• The recall time frame of typical health care cost items should not exceed three to four months, although longer periods are possible for specific items depending on their salience (hospital admissions, long-term sick leave) [9,18,25,26].

• Closed questions should be used if possible in order to support the effects of recognition [27].

Based on this information, the cost items in the questionnaire were divided according to their salience and their regularity of occurrence. The first part comprises cost components and health care utilization items that occur regularly and tend to be relatively unimportant to the patient. Items with these characteristics include the services of professional or informal caregivers, travel expenses, and additional costs for physical activities related to heart disease (table 1). The decision which variables would satisfy these conditions has been based on plausibility and patient opinion within the asked patient group. The second part comprised all items that occur regularly but tend to be more important to patients.

**Table 1:** Cost components measure in cost questionnaire

**Cost component**	**Parameter**	**Recall period**	Valuation*
Utilization of paid and unpaid help	Nature of help, duration (hours), outlays	4 weeks	Mean gross wage of a housekeeper
Travel expenses	Type of transportation, number of rides, cost (if public transportation) or kilometers (car)	4 weeks	Outlays (public transportation), official schedule of travel expenses (car)
Physical activities, gym activities	Type of activity, average number per week, duration, outlays	4 weeks	Average prices per activity
Physician services	Specialization of physician, number of consultations, services performed	12 weeks	Mean reimbursement per consultation
Intake of prescribed medication	Name of medication, current daily dosage, changes in medication or dosage	12 weeks	Official pharmaceutical price list
Intake of unprescribed medication	Name of medicine, number and size of packages purchased	12 weeks	Official pharmaceutical price list
Physical therapeutics/non-physician services	Type and number of services	12 weeks	Pricing schedule of statutory health insurance
Alternative care	Type and number of services, outlays	12 weeks	Outlays or price schedule of alternative care
Medical products	Type of product, outlays/cost	12 weeks	Average market prices
Change in diet	Type of dietary changes, estimated additional outlays	12 weeks	Outlays estimated by patients
Lost productive time	Reason for lost productive time, duration	12 weeks	Average sex- and age-adjusted labor costs
Inpatient stay	Reason for inpatient treatment, duration	12 weeks	Average per diem rates**

*Valuation in 2006 prices following suggested unit cost [33].

** Mean department specific hospital cost per day as suggested by Krauth et al. [33].

Ingested medications were a mixed case. Recommended medications for heart disease are similar in the various guidelines, and their administration is typically regular and over the long term. Substitutions between various drugs within the same class may occur periodically [28-30]. Furthermore medication rates have generally been observed to remain quite stable after an ACE [31,32]. However, changes in prescribed medications tend to be related to an acute event, which might be more important to the patient. This was resolved by asking the patient for their current daily intake. A subsequent question asked for changes in drug prescriptions occurring in the last three months. Correct recall was supported by "bounded recall" techniques. Thus, for the 3-month questionnaire, patients received a list with the discharge medications from cardiac rehabilitation.

The ordering of the questions was driven by the common recommendation in questionnaire design to start with rather easier questions e.g. [11] as well as the aim to avoid participants' confusion by switching between reference periods more often than needed. The questionnaire is available in its original German version as well as in an English translation which, however, has not been formally assessed [see Additional files 1 and 2].

Costs for medication and medical aids were valued using standard prices [33]. Reported days off work and hospital days were multiplied by average labour costs and hospital costs respectively. All costs are inflation adjusted and presented in 2006 Euros.

### Practicability/acceptability

The feasibility and acceptance of a cost questionnaire are crucial for its use in health economic studies. This is assessed by the response rate, which is defined as the proportion of returned questionnaires. As filter questions were used for each item, missing entries were defined as either missing information on the filter question itself or missing further information if the filter question was positively checked. Acceptance was also assessed by asking participants to rate the degree of difficulty of filling in the questionnaire on a four-point Likert scale.

### Completeness and face validity

In order to determine the relevant resource categories related to cardiac problems, eight patients who underwent rehabilitation care after ACE were interviewed about their typical medical consumption and other disease-specific non-medical utilization. They were also asked to rate the relevance of the different cost items by themselves. This was used to differentiate between questions in a 4-week and 3-month recall period. Face validity of the questionnaire was then assessed by medical experts consisting of a physician at the rehabilitation hospital and two outpatient physicians. They judged the completeness and consistency of the questionnaire draft.

### Convergent validity

Convergent validity was tested by comparing the information provided by the questionnaire with an alternative collection method of patient reported data – a prospective cost diary – which was based on previously tested and reported instruments [5,13]. Patients completed the cost diary prospectively over 4 weeks after cardiac rehabilitation. The information from the prospective cost diary was compared with the information from the retrospective questionnaire for an overlapping time period of 4 weeks. As some cost items were retrospectively assessed for the last 4 weeks (table 1), comparisons were possible for these cost items. Additional comparisons were possible by matching data which were asked in date format (hospital spells, spell off work), by reconstructing the retrospectively given history of medical intake, and by asking the patients in the tested questionnaire to assign the stated physician visits within the last 12 weeks to one of three 4 week spells after discharge from rehabilitation hospital.

### Criterion validity

For the cost items, days off work and hospital days, a third "objective" assessment was possible using administrative data from the statutory sickness funds which constitute the German public health insurance covering ~90% of the German general population. However, this analysis was only performed on a sub-sample, as some patients refused to allow the sickness fund the release of their data (n = 29), not all sickness funds responded to our request (n = 5), and some patients were privately insured (n = 11). Overall, the sickness funds provided information for 48 patients.

If patients consented, sickness funds were asked to list all sick leave and hospitalization days with the respective diagnoses codes during the 3 months after rehabilitation treatment (reference period). The validity of the questionnaire was assessed according to these data, which were considered to be the gold standard because sickness funds must pay sick benefits and hospital charges based on these data.

### Study design

On admission to the rehabilitation hospital in southern Germany, 106 consecutive patients requiring rehabilitation (3–4 weeks) after an ACE were recruited. Exclusion criteria were (as in the subsequent clinical trial) age above 65 years, insufficient command of the German language, and an ACE occurring more than 3 months before admission to the rehabilitation hospital.

Patients received the prospective 4-week cost diary upon discharge from the rehabilitation hospital, while the 3-month retrospective cost questionnaire was mailed 3 months after discharge. Patients were instructed to complete the questionnaire by themselves.

If necessary, patients were mailed two reminders and, if they still did not return the questionnaire, they were telephoned. Participants also received a gift coupon of 12 € as an incentive after completing and returning the questionnaire. Monetary incentives have been shown to increase response rates for health questionnaires [34].

### Statistical analysis

Internal consistency was tested using contingent tables with categorically scaled data. Intraclass correlation coefficients (ICC) between the prospective 4-week cost diary and the retrospective cost questionnaire were calculated from metrically scaled data, as this coefficient is a more suitable measure of agreement than, for example, Pearson correlation [33]. To test validity, retrospective self-reports and administrative data were compared by t-test and intraclass correlation. Self-reported retrospective data and administrative data were also compared by plotting the differences between the values against the mean of the values in order to test for a relationship between the difference and the magnitude of the two values [35].

Calculations and statistical analysis were performed using the software package SAS 8.02 (SAS Institute Inc., Cary, NC, USA).

### Ethics

The study was approved by the Ethics Committee of the State Medical Chamber of Baden-Württemberg (Landesärztekammer). Informed written consent was obtained from each individual enrolled into the study.

## Results

### Patients

The sociodemographic and clinical characteristics of the 106 patients enrolled in the study are shown in table 2. The sample reflects the typical sex distribution of patients after an acute cardiac event which is dominated by males.

**Table 2:** Participant characteristics (n = 106)

*Age *(*years*)		*Insurance status*	
Mean (SD)	55 (7.6)	Statutory health insurance	91 (86%)
Range	30–65	Private health insurance	13 (12%)
*Sex*		n.a.	2 (2%)
Male	90 (85%)	*Diagnosis*	
Female	16 (15%)	Myocardial infarction	54 (51%)
*Vocational education*		CABG	45 (42%)
No	4 (4%)	Angina	7 (7%)
Vocational training	52 (49%)	*Duration of disease since diagnosis (months)*	
Technical school	23 (22%)		
University/technical college	21 (20%)	Mean (SD)	20.6 (47.7)
Other/n.a.	6 (6%)	Range	0.5–264
*Persons in household*		*NYHA classification*	
Mean (SD)	2,7 (1,2)	class I	88 (83%)
Range	1–6	class II	10 (9%)
*Employment status*		class III	3 (3%)
Full time	66 (62%)	class IV	0
Less than full time	4 (9%)	n.a.	5(5%)
Unemployed	3 (3%)	*Self-rated health 3 months after rehabilitation (VAS)*	
Early retirement	9 (8%)		
Regular retirement	16 (15%)	Mean (SD)	78.6 (15.8)
Other/n.a.	1 (1%)	Range	30–100

NYHA, New York Heart Association range: class I = light limitations, class IV = inability; CABG, coronary artery bypass graft surgery; n.a., not available; VAS, visual analogue scale; range: 0 (= worst) to 100 (= best).

### Feasibility and acceptability

The total response rate of the cost questionnaire was 88% (93/106). Sixty per cent (57) of the sample returned the questionnaire without an additional reminder. Roughly one-fifth (21) of respondents reacted after the first and another 14% (13) after the second reminder letter. Two participants only responded after being called. The 13 non-respondents were on average 4.8 years younger than the respondents (p = 0.04) and were more likely to be employed full time than respondents (60% vs. 77%).

The 20 filter questions, which had to be checked in each questionnaire, were used as an indicator of the degree of completion of the questionnaire. From the total of 1860 (20 × 93) questions to be answered by the 93 responders, 15 (0.8%) questions were not filled in. In addition the sequence accuracy after the filter question was assessed. In just two cases a positively checked filter question was not followed by a meaningful entry, whereas after all negatively checked questions the following detail table remained (rightly) blank.

Most of the respondents (92%) found the questionnaire easy or very easy to answer. Information on the average duration of completion was provided by 88 respondents. Time for completion was on average 27 minutes (standard deviation (SD) 25.6, ranging from 4 to 180 minutes, median 20). For 85% of the sample, completion of the questionnaire did not take more than 30 minutes.

### Convergent validity

Convergent validity of patient information was studied by comparing the information of the retrospective cost questionnaire with a prospective diary. The main result of the comparison is shown in table 3. The ICCs of the compared cost items ranged between 0.57 and 0.90. Except for medications, the retrospectively measured mean values of health care utilization tended to be lower than the prospective measurements.

**Table 3:** Comparison of prospective cost diary and retrospective questionnaire for overlapping time (n = 90)*

	**Retrospective cost questionnaire**	**Cost diary**	**ICC**	p**
	Mean (SD)	Mean (SD)		

Number of physician visits	1.9 (1.9)	2.2 (2.2)	0.64	0.13
Inpatient days	0.54 (2.1)	0.54 (2.0)	0.90	0.99
Days off work	13.8 (14.9)	15.7 (14.0)	0.77	0.06
Number of drugs	4.1 (1.5)	3.9 (1.4)	0.72	0.09
Cost of medical intake (€)	2.9 (1.5)	2.6 (1.3)	0.57	0.04
Cost of medical aids (€)	162.3 (258.6)	175.5 (264.6)	0.72	0.52

*For three participants, comparison was not feasible as they did not return their cost diaries.

**Wilcoxon sign rank test.

A more detailed analysis of the medications is provided in table 4. While the names of the products agreed in 61% of the cases, there was a higher rate of agreement when the medical agents themselves were analyzed (81%).

**Table 4:** Comparison of medications measured by cost diary and retrospective questionnaire (n = 90)*

Reliability analysis		Medication (sales name)	Medication (generic name/agent)
Questionnaire	Diary		
Yes	Yes	274 (61.3%)	325 (80.8%)
Yes	No	77 (17.2%)	28 (7.0%)
No	Yes	95 (21.3%)	48 (11.9%)
No	No	1** (0.2%)	1** (0.2%)

* For three participants, comparison was not feasible as they did not return their cost diaries.

**Patients who did not report any medical intake.

### Criterion validity

**Figure 1:**
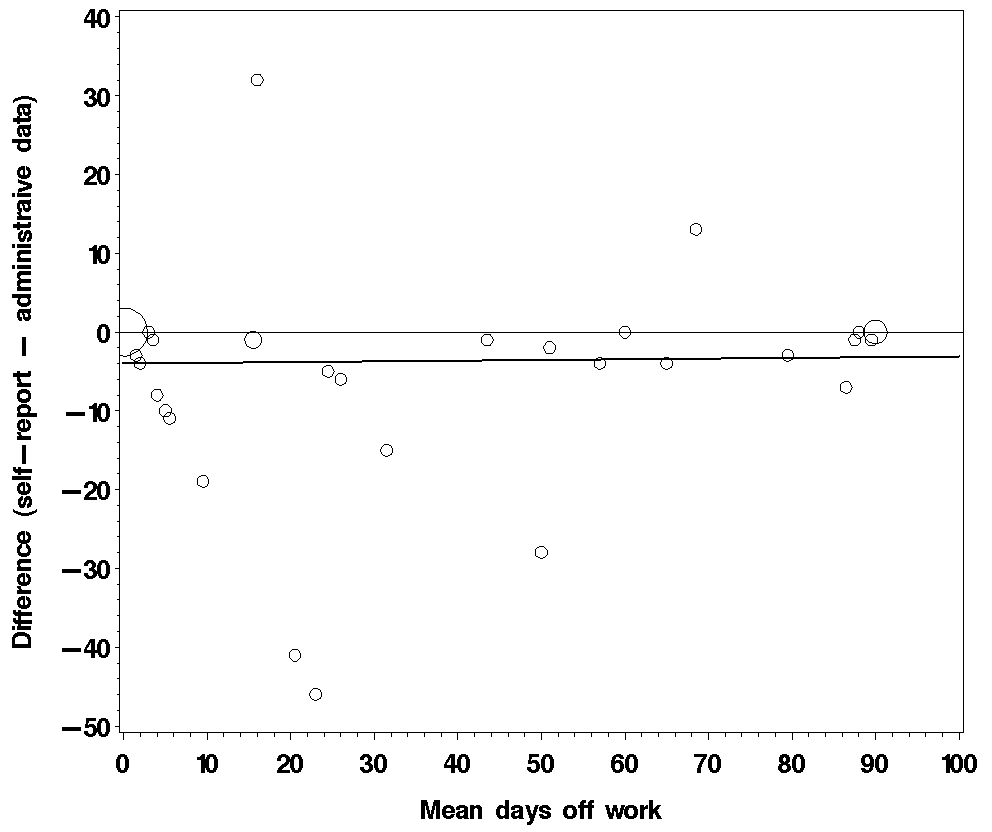
**Bubble plot of difference (self-report–administrative data) by mean days off work (n = 48)**. The bold solid line represents the regression line; the thin line is drawn at zero representing exact agreement. The bubble size is proportionate to the number of patients with corresponding values.

The outcome of the validity analysis comparing the 3-month patient questionnaire and the sickness fund data is reported in table 5. The intraclass correlations, used as a measure of agreement, are generally well above 0.7, which indicates good agreement. However, the paired t-test reveals significant differences between the mean values of sick leave, which leads to an underestimation of the indirect costs. Figure 1 illustrates the differences plotted against the mean of patient-provided data and administrative data. The graph shows that underreporting did not increase with increasing sick leave days.

**Table 5:** Comparison of questionnaire and sickness fund data (n = 48)

	**Retrospective ** **cost questionnaire**	**Sickness fund** ** data**	**Zero difference ** **(+/- 1)**	**ICC**	p*
	
	Mean (SD)	Mean (SD)	n (%)		
Days off work hospitalized	1.8 (7.45)	1.8 (7.5)	42 (87.5)	0.92	0.92
Days off work not hospitalized	25.3 (33.4)	29.1 (33.5)	25 (52.1)	0.94	0.03
Total days of sick leave	27.2 (35.2)	30.9 (34.9)	29 (60.4)	0.94	0.03

*Wilcoxon sign rank test.

Based on the full sample (n = 90), patients reported more sick leave in the questionnaire than in the diary. However, in the subpopulation for which sickness fund data were available (n = 48), subjects underreported sick leave days in the questionnaires over the 3-month period. Comparing the 4-week period covered by the available sickness fund data and the diary showed that the mean sick leave days agreed well between the diaries and the sickness fund data (17.1 vs. 17.6 days, p = 0.60), whereas there was slight underreporting of sick leave in the questionnaire (16.3 days, p = 0.24).

### Health care and disease-related costs

The results of the cost measurement analysis are shown in table 6. As expected, the indirect costs accounted for the major part of the disease-related costs. Among the direct medical costs, hospitalization accounted for most of the costs followed by the prescribed drugs, reflecting the great relevance of pharmaceutical treatment in patients after ACE. Most of the direct cost items are skewed to the right, typical of health care costs. Indirect and, therefore, total costs are minimally skewed, but instead have a u-shaped distribution because of a significant proportion of patients with minimum or maximum indirect costs.

**Table 6:** Cost of care (for 3 months) based on retrospective data (n = 93)

	**Percent **	**Mean**	**SD**	**Range**	**Skewness**
Direct medical cost*	*13.1%*	*1,050.5*	*1,767.5*	*83.9–14070.8*	*5.24*
Hospitalization	43.5%	457.0	1714.7	0.0–13,865.6	6.07
Prescription drugs	23.8%	250.3	119.6	26.9–778.6	1.27
Medical aids	18.9%	198.5	408.0	0.0–3,236.4	5.02
Physician	12.7%	133.0	232.4	0.0–1726.0	5.12
Non-prescription drug	0.8%	8.1	51.1	0.0–470.7	8.45
Courses	0.2%	2.0	11.2	0.0–77.2	5.74

Alternative care	0.1%	1.5	9.2	0.0–76.7	7.08

Non-medical cost*	*2.6%*	212.2	357.7	*0.0–2,112.4*	3.52

Services (paid/unpaid)	52.4%	111.2	341.1	0.0–2,036.9	4.08

Activities	20.3%	43.2	41.8	0.0–196.2	0.56
Additional cost for diet	18.7%	39.6	87.9	0.0–385.8	2.40
Travel expenses	8.6%	18.2	40.9	0.0–277.8	3.76

Indirect cost*	*84.2%*	6,754.0	8,433.5	*0.0–2,1673.7*	0.77

Time off work for physician visits	0.3%	18.6	53.8	0.0–273.4	3.10
Time off work	99.7%	6,735.5	8,434.9	0.0–21,804.5	0.77

Total cost	100.0%	8,016.8	9,122.5	83.9–34,522.0	0.85

*Percentage of total cost.

## Discussion

Health economic studies incorporating a societal perspective and performed alongside clinical trials must rely at least partially on patient-based information on resource consumption. This study's aim was to describe the development of a questionnaire measuring direct and indirect costs in patients after an acute cardiac event and to test its properties in terms of feasibility and aspects of validity.

### Study design

The questionnaire was developed based on the results of survey research and cognitive psychology regarding the appropriate time frame of the cost items required to completely assess costs in an economic evaluation. In the development of this retrospective questionnaire, the major issues concerning the accuracy of utilization measurement, which were summarized recently by Bhandari and Wagner, were considered: the questionnaire was developed specifically for the sample population and their utilization patterns, recall aspects were taken into account, different forms of data collection were compared, and the instrument was finally revised with improved instructions [15]. By explicitly describing the development process and the validation, we seek to contribute to the standardization of cost instruments and their standardized validation.

### Acceptability and feasibility

The questionnaire was easy to use and showed a high response rate of 88%. The average completion time of 27 minutes is at the upper time limit acceptable to patients [36]. As the median is considerably lower (20 minutes) and only 15% of subjects required more than 30 minutes for completion, the time burden was probably acceptable for most subjects. However, this could be a practical research problem if other patient-oriented outcomes are measured in addition to costs. As cost measurement showed that a substantial percentage of costs are due to just a few main cost drivers such as days off work, hospital admissions, and medications, it would be possible to reduce the number of cost items and related utilization questions without losing too much relevant information. However, this must be decided specifically for each study as there is a trade off between level of detail, accuracy and completeness versus acceptability.

The response pattern indicates the usefulness of reminders. Even the second reminder letter substantially raised the response rate whereas a third contact by telephone did not show a further significant improvement in participation. A third reminder letter is also probably useless because, by the time a third letter is sent, the likelihood of recall error and telescoping effects increases.

A problem in the study was the selection bias of responders. The age difference between responders and non-responders was 5 years, and the percentage of non-responders in full-time employment was substantially higher. Comparable studies on patient-reported utilization did not find differences in compliance with respect to age but with respect to marital status [5]. The lower response rate of patients in full-time employment may be due to their tighter time schedule. Thus, the length of the questionnaire should be as short as possible, and the importance of continued response should be thoroughly explained to patients when motivating them to participate in the study. In clinical trials, results should be checked for an influence of full-time employment status.

### Assessment of validity

Our comparison of the questionnaire with a patient diary and with data from sickness funds regarding some key variables supports its validity for cost measurement. The questionnaire agreed reasonably with the patient diary for variables that could be compared over the time period of 4 weeks. Comparison of medications showed that 61% agreed in terms of names and 81% agreed with respect to the active agent. Comparison of hospitalization, sick leave, and physician visits showed moderate to good correlation, although a significant difference in the level was observed. There was a tendency to underreporting compared with the prospective data and the administrative data, which has often been reported in studies analyzing retrospective patient-reported data compared with administrative or provider data [4,5,12,37,38].

In particular, the underreporting of sick leave and, therefore, indirect costs must be noted as this cost component is the dominant cost driver. This corresponds with other recent studies that have also found underreporting of lost productive time [9,38]. One reason for this underreporting seems to be partly a lower response rate in patients who are active in the labor force. Therefore, measures aimed at increasing the response rate of working patients might lower the extent of underreporting in this important cost component.

A detailed analysis showed that the major difference in the mean value was effectively caused by single cases for which there was a great discrepancy between self-reported and administration data. It seemed that patients who were jobless at the time of the cost survey did not record sick leave although they were (correctly) listed in the sick leave data. Thus, describing this case in the instructions accompanying the questionnaire may increase agreement between the two sources.

Despite the good concordance indicated by measures of (linear) agreement, absolute utilization was mostly underreported when the results of the questionnaire were compared with administrative data and the diary. As this tendency does not appear to depend on baseline characteristics or health status, it seems unlikely that such deviations could bias the cost measurement of disease-related costs when differences between treatment groups are analyzed, as is usually the case in health economic evaluation studies.

As expected, the disease-related costs measured by this questionnaire are dominated by high indirect costs, which are known to be an important consequence of heart disease [39]. The information on the distribution and variance in the data provides valuable information for statistical planning of health economic studies. Although the questionnaire has been developed to cover the typical utilization patterns of patients after an ACE, it could be adapted to other diseases, including layout and instructions, with minimal effort.

### Limitations

There were limitations of this study which need to be mentioned. As ACE has a high incidence in age groups over 65 years, the findings are not totally representative for the unrestricted population. No significant age-related differences were found in the quality of reporting in our study. This corresponds with the findings of Ritter et al., who did not detect any reporting differences according to demographic characteristics [4]. Additional validation research focusing also on higher age classes would be of high interest.

The size of the study was planned to indicate whether the measurement approach was feasible and reasonably valid. For more detailed analysis, a larger study including a comparison with administrative data regarding medication and physician visits would be desirable. Due to various reasons, the comparison of the questionnaire with administrative data was just possible for n = 48 representing 45% of the starting sample of 106, which is a significant loss in power and might induce bias. The described selection process implies that for the n = 29 patients who did not sign the related patient agreement a systemic bias cannot be fully excluded, although there is no intuitive assumption as to how and for what reason the response pattern and agreement would systematically vary with the fact that the participants disagreed to contact. The information for the other cases n = 16 is in technical terms missing at random and therefore unlikely to introduce any bias for the results of the remaining sample.

As health care utilization is potentially subject to treatment variability, generalizability across countries cannot be taken for granted. In the case of secondary prevention after ACE, comparable treatment patterns are suggested across countries [28,30], and thus the basic structure of potential utilization should be comparable between countries. Differences in resource availability and accessibility to health care systems will mainly impact the frequency of utilization (e.g. number of physician visits or special procedures in hospital), which can then be captured by the questionnaire.

The severity of the disease state may potentially have an impact on questionnaire acceptance. Considering the disease classification according to the New York Heart Association (NYHA) and self-rated health, the sample in this study was moderately affected by the consequences of their ACE. As there was only a weak correlation between measures of disease severity and questionnaire acceptance, this provides some evidence that the questionnaire can also be used in more severely affected populations as long as patients are cognitively and physically capable of completing the forms.

Also asking for information in overlapping periods might have had a positive impact on patients' commitment and their thoroughness in completing the questionnaire, resulting in higher acceptance and questionnaire agreement than in a non-clinical trial setting. This effect cannot be excluded for every research dealing with repeated measurements and might be dampened in this case by the fact that in most cases a time span of 8 weeks was in between the administration of the two instruments.

A final limitation of this questionnaire is that it is targeted on cardiac patients. It should be noted that before applying instruments to other areas of diseases a similar process of adaptation is recommended in order to capture the disease specific health care utilization. This process could include a literature review of main treatment patterns and examination and assessment of the draft instrument by medical experts as well as patients.

## Conclusion

With the population under study the questionnaire was well accepted and showed good correlation with alternative measurement approaches. This preliminary assessment showed some promising characteristics of this questionnaire in different aspects of validity for patients with ACE and might present a possible alternative for the measurement of study costs in settings where the collection of administrative data is not feasible. However, additional research and more extensive tests would be worthwhile.

## Competing interests

The authors declare that they have no competing interests.

## Authors' contributions

BS participated in the design of the study, performed the statistical analysis and drafted the manuscript. HH participated in the design of the study, organized the data collection and reviewed the manuscript. RL conceived the study, participated in its design and coordination and helped to draft the manuscript. All authors read and approved the final manuscript.

## Pre-publication history

The pre-publication history for this paper can be accessed here:



## Supplementary Material

Additional file 1 Cost questionnaire BMC German (Kostenbuch). Original German version of the tested cost questionnaire. Click here for file

Additional file 2 Cost questionnaire BMC English (Cost Diary). Straightforward translation of the original German version (additional file 1). See also disclaiming remarks before using it. Click here for file
