# Supplementary material for: Development and first assessment of a questionnaire for health care utilization and costs for cardiac patients
Source: BMC Health Serv Res. 2008 Sep 19;8:187. doi: 10.1186/1472-6963-8-187 (PMC2556330; doi:10.1186/1472-6963-8-187)
Supplement: Additional file 1 — Cost questionnaire BMC German (Kostenbuch). Original German version of the tested cost questionnaire. [file 1472-6963-8-187-S1.pdf]

Kostenbuch

Was kostet Ihre Krankheit wirklich?

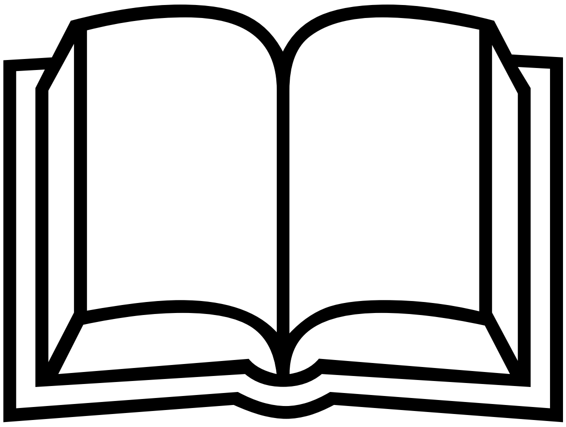

Datum: ______________________________

Name: ______________________________

Adresse: ______________________________

____

Ulmer Rehaverbund: Pilotstudie kardiologische Rehabilitation
Klinik Schwabenland und Abteilung Gesundheitsökonomie, Universität Ulm

Anleitung zum Ausfüllen des Kostenbuchs

Bitte füllen Sie dieses Buch 3 Monate nach Ihrer Entlassung aus der Schwabenlandklinik aus.

Bitte beachten Sie beim Ausfüllen, dass für diese Studie <u>nur</u> Ausgaben und Leistungen, die mit Ihren Herzbeschwerden zusammenhängen, von Bedeutung sind. Sollten Sie zum Beispiel wegen einer Grippe einen Arzt aufgesucht und ein Arzneimittel verschrieben bekommen haben, tragen Sie das bitte nicht in dieses Kostenbuch ein.

Gehen Sie bitte Schritt für Schritt in aller Ruhe die folgenden Seiten durch. Auch wenn Ihnen unser Kostenbuch auf den ersten Blick umfangreich erscheinen mag, werden Sie nicht viel Zeit für das Beantworten der Fragen benötigen. Es ist sehr unwahrscheinlich, dass Sie zu allen Fragen Angaben machen können.

Beantworten Sie bitte jede Frage. Trifft **„Nein“** zu, können Sie direkt zur nächsten Frage übergehen. Trifft **„Ja“** zu, füllen Sie bitte auch die zugehörige Tabelle aus.
Sie können dort mehrere Angaben untereinander machen, z.B. wenn Sie mehrere Medikamente eingenommen haben.

Manchmal werden Sie gebeten, zu einem Sachverhalt mehrere Angaben zu machen. Wenn Sie zum Beispiel Hilfe von Familienangehörigen in Anspruch genommen haben tragen Sie bitte die angefallene Zeit ein. Sollten Ihnen daneben auch noch Kosten entstanden sein tragen Sie diese ebenfalls in die Tabelle ein.

Beispiel:

**Hilfen**<br>(Art der Hilfe)	Durchschnittlicher Zeitaufwand und Kosten pro Woche
Hilfe von Familienangehörigen, Freunden oder Bekannten	Stunden: 4
	€: 20,-

Bitte gehen Sie auf diese Weise Frage für Frage durch. Beachten Sie, dass manchmal anstatt nach Anzahl der Kontakte oder Kosten auch nach anderen Angaben gefragt wird.

Auf der letzten Seite finden Sie noch einige Antworten auf Fragen, die sich Ihnen beim Ausfüllen des Kostenbuchs stellen könnten.

Wenn Sie das Kostenbuch ausgefüllt haben, schicken Sie es bitte im beiliegenden frankierten Umschlag zurück an die Klinik Schwabenland.

Wir versichern Ihnen, dass wir mit Ihren Angaben vertraulich umgehen.

Als Dankeschön für Ihre Mühe erhalten Sie für die <u>beiden</u> von Ihnen ausgefüllten und zurückgesandten Kostenbücher einen **Einkaufsgutschein im Wert von 12€**.

Zur Beachtung:

- Bitte machen Sie nur Angaben, die im Zusammenhang mit Ihren Herzbeschwerden stehen.
- Bitte schicken Sie das Kostenbuch im beiliegenden und frankierten Rückumschlag an die Klinik zurück.

Sehr geehrte Studienteilnehmerin, sehr geehrter Studienteilnehmer bitte beachten Sie:

Die folgenden Fragen betreffen den Zeitraum

der letzten 4 Wochen

vor 4 Wochen	vor 3 Wochen	vor 2 Wochen	letzte Woche
Mo	Mo	Mo	Mo
Di	Di	Di	Di
Mi	Mi	Mi	Mi
Do	Do	Do	Do
Fr	Fr	Fr	Fr
Sa	Sa	Sa	Sa
So	So	So	So

Zur besseren Veranschaulichung des betreffenden Zeitraums haben wir für Sie mit blauem Stift auf dem beigelegten Kalenderblatt ausgehend vom _____________ die letzten 4 Wochen markiert.

1. Haben Sie wegen Ihrer Herzbeschwerden in den letzten 4 Wochen **Hilfe von Familienangehörigen, Freunden, Bekannten oder professioneller Seite** für Arbeiten in Anspruch nehmen müssen, die Sie üblicherweise selber erledigen (z.B. um den Haushalt oder Einkäufe zu erledigen)?

Nein ☐ **Ja** ☐

Wenn „**Ja**“, füllen Sie bitte die folgende Tabelle 1 aus, sollten Sie **„Nein“** angekreuzt haben, gehen Sie bitte zu Frage 2 über.

Tabelle 1

Bitte tragen Sie hier den durchschnittlichen Zeitaufwand in Stunden und die durchschnittlichen Kosten der Hilfen pro Woche ein. Sollten Sie die Kosten nicht wissen, tragen Sie bitte ein Fragezeichen ein.

Art der Hilfen	Durchschnittlicher Zeitaufwand pro Woche	Durchschnittliche Kosten pro Woche
Hilfe von Familienangehörigen, Freunden oder Bekannten	|______| Std.	|______| €
Haushaltshilfen	|______| Std.	|______| €
Ambulante Pflegedienste (z.B.: Caritas)	|______| Std.	|______| €
	|______| Std.	|______| €
	|______| Std.	|______| €
	|______| Std.	|______| €

2. Haben Sie in den letzten 4 Wochen aufgrund Ihrer Herzbeschwerden Ihren **Privatwagen** oder **öffentliche Verkehrsmittel** zu Fahrten benutzt, die Sie ohne Ihre Erkrankung nicht unternommen hätten (z.B., um zu Ihrem Arzt zu kommen)?

Nein ❑ **Ja** ❑

Wenn „**Ja**“, füllen Sie bitte die folgende Tabelle 2 aus, sollten Sie **„Nein“** angekreuzt haben, gehen Sie bitte zu Frage 3 über.

Tabelle 2

Bitte tragen Sie hier die Anzahl der Kilometer ein, die Sie mit Ihrem Auto gefahren sind, beziehungsweise die Kosten, die Ihnen entstanden sind, falls Sie andere Verkehrsmittel benutzt haben. Sollten Sie diese nicht wissen, tragen Sie bitte ein Fragezeichen ein.

Verkehrsmittel (Art)	Anzahl der Fahrten (Hin- und Rückfahrt entspricht einer Fahrt)	Kilometer oder Kosten der Fahrten insgesamt
PKW	|_______|	|_______| km
Straßenbahn/Bus (Kosten der Fahrkarten)	|_______|	|_______| €
Zug (Kosten der Fahrkarten)	|_______|	|_______| €
Taxi (Kosten der Fahrten)	|_______|	|_______| €
	|_______|	|_______| km/€
	|_______|	|_______| km/€

3. Sind Sie in den letzten 4 Wochen zur **Vorbeugung oder Linderung ihrer Herzbeschwerden** regelmäßig aktiv geworden, indem Sie z.B. in einer Herzsportgruppe mitgemacht haben, oder sind Sie deswegen zum Radfahren oder ins Fitnessstudio gegangen?

Nein ☐ **Ja** ☐

Wenn „**Ja**" , füllen Sie bitte die folgende Tabelle 3 aus, sollten Sie **„Nein"** angekreuzt haben, gehen Sie bitte zum zweiten Teil des Fragebogens über.

Tabelle 3

Bitte tragen Sie hier die Anzahl der Aktivitäten ein. In der dritten Spalte geben Sie bitte Ihre Einschätzung an, ob diese Anzahl im Vergleich zu den vorangegangenen 2 Monaten weniger, gleich viel oder mehr an Aktivitäten entspricht.

Aktivitäten	Durchschnittliche Anzahl der Aktivitäten pro Woche	Im Vergleich zu den vorangegangenen 2 Monaten		
		weniger	gleich viel	mehr
Herzsportgruppe	|______|	☐	☐	☐
Radfahren	|______|	☐	☐	☐
Fitnessstudio	|______|	☐	☐	☐
Schwimmen	|______|	☐	☐	☐
	|______|	☐	☐	☐
	|______|	☐	☐	☐
	|______|	☐	☐	☐
	|______|	☐	☐	☐

Sehr geehrte Studienteilnehmerin, sehr geehrter Studienteilnehmer bitte beachten Sie:

Die folgenden Fragen betreffen den Zeitraum

der letzten 3 Monate

vor 3 Monaten	vor 2 Monaten	letzter Monat
1.	1.	1.
2.	2.	2.
3.	3.	3.
..	..	..
..	..	..
..	..	..
29.	29.	29.
30.	30.	30.
31.		31.

Zur besseren Veranschaulichung haben wir für Sie mit gelbem Stift auf dem beigelegten Kalenderblatt den betreffenden Zeitraum der letzten 3 Monate vom ______________ bis zum ______________ markiert.

1. Haben Sie in den letzten 3 Monaten einen **Krankengymnasten, Physiotherapeuten, Heilpraktiker oder anderen Therapeuten** aufgesucht? Wenn ja, welche Leistungen haben Sie dort in Anspruch genommen (z.B. Bewegungstherapie)?

Nein ☐ **Ja** ☐

Wenn „**Ja**“, füllen Sie bitte die folgende Tabelle 1 aus, sollten Sie **„Nein“** angekreuzt haben, gehen Sie bitte zu Frage 2 über.

Tabelle 1

**Therapeut** (Fachrichtung)	Leistungen (bitte kurz beschreiben)	Anzahl der Leistungen	Kosten aller Besuche (in €)
Krankengymnast	1. ____________<br>2. ____________	|_____| |_____|	|_____| |_____|
Physiotherapeut	1. ____________<br>2. ____________	|_____| |_____|	|_____| |_____|
Heilpraktiker	1. ____________<br>2. ____________	|_____| |_____|	|_____| |_____|
	1. ____________<br>2. ____________	|_____| |_____|	|_____| |_____|
	1. ____________<br>2. ____________	|_____| |_____|	|_____| |_____|

2. Haben Sie in den letzten 3 Monaten aufgrund von Herzbeschwerden einen Arzt (z.B. Ihren **Hausarzt, Kardiologen oder Psychologen)** aufsuchen müssen (auch Telefonate) oder einen Hausbesuch bestellen müssen? Wenn ja, welche Leistungen haben Sie erhalten (z.B. EKG, Blutuntersuchung, Röntgen, Ultraschall oder Rezept abgeholt)?

Nein ☐ **Ja** ☐

Wenn „**Ja**", füllen Sie bitte die folgende Tabelle 2 aus, sollten Sie **„Nein"** angekreuzt haben, gehen Sie bitte zu Frage 3 über.

Tabelle 2

**Haus- und Facharztbesuche** (Fachrichtung)	Anzahl der Kontakte in den letzten 3 Monaten	Was wurde gemacht ?	Wie oft?
Hausarzt	|_____|	☐ EKG<br>☐ Blutuntersuchung<br>☐ Röntgen<br>☐ Ultraschall<br>☐ Rezept abgeholt<br>☐ Telefonat<br>☐ _______________	|_____| |_____| |_____| |_____| |_____| |_____| |_____|
Internist/ Kardiologe	|_____|	☐ EKG<br>☐ Blutuntersuchung<br>☐ Röntgen<br>☐ Ultraschall<br>☐ Rezept abgeholt<br>☐ Telefonat<br>☐ _______________	|_____| |_____| |_____| |_____| |_____| |_____| |_____|
Psychologe	|_____|	☐ Beratung<br>☐ _______________	|_____| |_____|
	|_____|	☐ _______________	|_____|
Ambulante Versorgung im Krankenhaus	|_____|	☐ _______________	|_____|

3. Waren Sie <u>in den letzten 3 Monaten</u> **während der Arbeitszeit** bei einem **Arzt oder Therapeuten**?

Nein ☐ **Ja** ☐

Wenn **„Ja“**, füllen Sie bitte die folgende Tabelle 3 aus, sollten Sie **„Nein“** angekreuzt haben, gehen Sie bitte zu Frage 4 über.

Tabelle 3

**Zeit, die Sie während der Arbeitszeit benötigten, um einen Arzt oder Therapeuten zu besuchen**	Gesamter Arbeitsausfall
Arztbesuche	|______| Std.
Therapeut	|______| Std.
	|______| Std.

4. Haben Sie <u>in den letzten 3 Monaten</u> **Veranstaltungen/Kurse**, die in direktem Zusammenhang mit Ihrer Erkrankung oder Ihren Herzbeschwerden stehen, besucht z.B. Informationsabende, Volkshochschulkurse über fettarme Ernährung, o. ä.)?

Nein ☐ **Ja** ☐

Wenn **„Ja“**, füllen Sie bitte die folgende Tabelle 4 aus, sollten Sie **„Nein“** angekreuzt haben, gehen Sie bitte zu Frage 5 über.

Tabelle 4

**Veranstaltungen/Kurse**	Kosten/Zeitraum
Beispiel:<br>*Autogenes Training*	|__40__| €/ <u>April – Mai 2002</u>
	|______| € ______________
	|______| € ______________
	|______| € ______________

5. Waren Sie <u>in den letzten 3 Monaten</u> aufgrund von Herzbeschwerden **krank geschrieben** (zu Hause oder im Krankenhaus)?

Nein ☐ **Ja** ☐

Wenn **„Ja“**, füllen Sie bitte die folgende Tabelle 5 aus, sollten Sie **„Nein“** angekreuzt haben, gehen Sie bitte zu Frage 6 über.

Tabelle 5

**Zeit, in der Sie krank geschrieben waren**	Datum von - bis	Datum von - bis	Datum von - bis
Krank geschrieben	von ___. ___. ___<br>bis ___ . ___ . ___	von ___. ___. ___<br>bis ___ . ___ . ___	von ___. ___. ___<br>bis ___ . ___ . ___
Verminderte Arbeits-zeit zur stufen-weisen Wieder-eingliederung	von ___. ___. ___<br>bis ___ . ___ . ___	von ___. ___. ___<br>bis ___ . ___ . ___	von ___. ___. ___<br>bis ___ . ___ . ___

6. Waren Sie in den letzten 3 Monaten aufgrund von Herzbeschwerden im **Krankenhaus** oder in einer **Rehaklinik**?

Nein ☐ **Ja** ☐

Wenn **„Ja"**, füllen Sie bitte die folgende Tabelle 6 aus, sollten Sie **„Nein"** angekreuzt haben, gehen Sie bitte zu Frage 7 über.

Tabelle 6

**KH-/Reha-Aufenthalte**	Datum von - bis	Datum von - bis	Datum von - bis
Krankenhaus-aufenthalte	von ___. ___. ___<br>bis ___ . ___ . ___	von ___. ___. ___<br>bis ___ . ___ . ___	von ___. ___. ___<br>bis ___ . ___ . ___
Grund der Aufnahme	________________	________________	________________
Maßnahme	☐ OP<br>☐ sonstiges:<br>________________	☐ OP<br>☐ sonstiges:<br>________________	☐ OP<br>☐ sonstiges:<br>________________
Reha-aufenthalte	von ___. ___. ___<br>bis ___ . ___ . ___	von ___. ___. ___<br>bis ___ . ___ . ___	von ___. ___. ___<br>bis ___ . ___ . ___

7. Haben Sie <u>in den letzten 3 Monaten</u> **Medikamente** gegen Ihre Herzbeschwerden oder Ihre Risikofaktoren (z.B.: Bluthochdruck, Cholesterin) eingenommen, die Ihnen vom **Arzt verordnet** wurden oder haben Sie **sich selbst Arzneimittel** gekauft?

Nein ☐ **Ja** ☐

Sollten Sie **„Ja“** angekreuzt haben, füllen Sie bitte die folgenden Tabellen 7a bis 7c aus, sollten Sie **„Nein“** angekreuzt haben, gehen Sie bitte zu Frage 8 über.

Tabelle 7a

Bitte tragen Sie hier die **verordneten Arzneimittel** ein, die Sie **zur Zeit** einnehmen. Wenn Sie das Medikament zur Hand nehmen, können Sie einfach die Angaben von der Packung übertragen. Bei Tabletten geben Sie bitte die Stückzahl und bei Tropfen die Anzahl der Tropfen pro Tag an.

**Arzneimittel auf Rezept** (Name)	Darreichungsform (Tabletten, Dragees, Tropfen, ...)	Verordnete Tagesdosis
Beispiel: Aspirin 100N	*Tabletten*	*1-0-1*

7b Hat sich bei Ihrer Medikation in den letzten 3 Monaten eine **Änderung** ergeben?

Zur Erinnerung haben wir Ihnen auf der beiliegenden Seite Ihre zuletzt eingenommenen Medikamente aufgelistet.

Nein ☐ **Ja** ☐

Wenn „Ja“ , bitte beschreiben Sie in der folgenden Tabelle die Art der eingetretenen Änderung

Tabelle 7b

**Arzneimittel** (Name+Darreichungsform)	Veränderung	Tagesdosis	Datum
Bsp:<br>*Aspirin 100N Tbl.*	☐ neu verordnet<br>☒ abgesetzt<br>☐ Dosis geändert	*1 – 0 – 1*	seit<br>*10.05.02*
oder:<br>*Aspirin 100N Tbl.*	☐ neu verordnet<br>☐ abgesetzt<br>☒ Dosis geändert	*von 1 – 1 – 1*<br>*auf 1 – 0 – 1*	seit<br>*10.05.02*
	☐ neu verordnet<br>☐ abgesetzt<br>☐ Dosis geändert		seit<br>___ . ___ . ___
	☐ neu verordnet<br>☐ abgesetzt<br>☐ Dosis geändert		seit<br>___ . ___ . ___
	☐ neu verordnet<br>☐ abgesetzt<br>☐ Dosis geändert		seit<br>___ . ___ . ___
	☐ neu verordnet<br>☐ abgesetzt<br>☐ Dosis geändert		seit<br>___ . ___ . ___
	☐ neu verordnet<br>☐ abgesetzt<br>☐ Dosis geändert		seit<br>___ . ___ . ___
	☐ neu verordnet<br>☐ abgesetzt<br>☐ Dosis geändert		seit<br>___ . ___ . ___
	☐ neu verordnet<br>☐ abgesetzt<br>☐ Dosis geändert		seit<br>___ . ___ . ___

Tabelle 7c

Bitte tragen Sie hier die **Arzneimittel** ein, die Sie sich aufgrund Ihrer Herzerkrankung **selbst** (ohne Rezept) gekauft haben. Sollten Sie die Kosten pro Packung nicht mehr wissen, tragen Sie bitte ein Fragezeichen ein.

Arzneimittel ohne Rezept (Name)	Anzahl der Packungen	Kosten pro Packung	Packungsgröße bitte ankreuzen		
			N1	N2	N3
	|_____|	|_____| €			
	|_____|	|_____| €			
	|_____|	|_____| €			
	|_____|	|_____| €			
	|_____|	|_____| €			
	|_____|	|_____| €			

8. Haben Sie sich z.B. in Zusammenhang mit Ihren Herzbeschwerden in den letzten 3 Monaten **Hilfsmittel** gekauft, z.B. Ergometer, Bücher, Blutdruckmessgerät? Oder haben Sie sogar umbauen müssen (z.B. einen Treppenlift einbauen)? Oder weitere Ausgaben tätigen müssen?

Nein ☐ **Ja** ☐

Wenn **„Ja“**, füllen Sie bitte die folgenden Tabellen 8a und 8b aus.

Tabelle 8a

Sollte eines der Beispiele in der Tabelle 8a zutreffen, kreuzen Sie es bitte an und tragen Sie den Preis ein. Bei weiteren Hilfsmitteln tragen Sie bitte die Bezeichnung und die Höhe der Ausgaben ein.

Hilfsmittel (Beispiele)	Kosten in €
☐ Ergometer<br>☐ Pulsmesser<br>☐ Blutdruckmesser	|______| |______| |______|
☐ Bücher<br>☐ Kassetten<br>☐ Videos	|______| |______| |______|
☐ Umbaumaßnahmen<br>☐ Sonstiges:<br>____________________	|______| |______|

Tabelle 8b

In der folgenden Tabelle 8b können Sie alle die Ausgaben eintragen, die Sie aufgrund Ihrer Erkrankung getätigt haben und die bisher noch nicht berücksichtigt wurden.

**Weitere Ausgaben, die bisher nicht aufgeführt wurden** (Bezeichnung)	Kosten in €
	|________|
	|________|
	|________|
	|________|

Antworten auf Fragen, die sich Ihnen beim Ausfüllen dieses Fragebogens vielleicht stellen

Ich habe eine Grippe gehabt und deswegen meinen Arzt besucht, der mich krank geschrieben hat. Muß ich jetzt alle Medikamente, den Arztbesuch, die Krankschreibung und die Zeit, in der ich meine Hausarbeit nicht erledigen konnte, eintragen?
Nein, bitte tragen Sie nur Dinge ein, die in direktem Zusammenhang mit Ihren Herz-beschwerden stehen. Alle Behandlungen und Aufwendungen, die Sie wegen anderer Krankheiten hatten, möchten wir nicht berücksichtigen.

Ich kann mich nicht mehr an die genauen Angaben auf der Packung meines Medikamentes erinnern. Was soll ich eintragen?
Bitte versuchen Sie die Angaben so genau wie möglich zu machen. Sollten Sie die alte Medikamentenpackung nicht mehr haben, um den Namen abzuschreiben, tragen Sie bitte die Art des Medikaments ein, z.B. Betablocker.

Ich habe die Rechnung meines Heilpraktikerbesuches noch nicht erhalten. Was mache ich jetzt?
Bitte tragen Sie ein Fragezeichen ein. Es sei denn, Sie sind sich aus Erfahrung schon ziemlich sicher wie hoch die Rechnung ausfallen wird. Dann tragen Sie bitte Ihren Schätzwert ein.

Es sind mir wegen meiner Herzbeschwerden Kosten entstanden, die ich nicht zuzuordnen weiß, da sie in keine Tabelle so recht zu passen scheinen. Wo soll ich diese eintragen?
Bitte tragen Sie alle Ausgaben, Behandlungen und Anwendungen, die Sie keiner Frage eindeutig zuordnen können, bei Tabelle 8b ein. Bitte vergessen Sie nichts, was mit Ihren Herzbeschwerden in Verbindung steht.

Zum Abschluss möchten wir Ihnen noch ein paar Fragen zu Ihrer Person und zu diesem Fragebogen stellen.

1. Haben Sie seit Ihrer Entlassung aus der Rehabilitation eine Umschulungsmaßnahme aufgenommen ?

❑ Ja ❑ Nein

Falls Sie **„Ja“** angekreuzt haben, bitte beantworten Sie folgende Fragen:

- Grund der Umschulung: ____

- Ziel der Umschulung: ____

- Zeitraum der Umschulung:

von: ____ / ____ / _______ bis: ____ / ____ / _______

2. Haben Sie seit Ihrer Entlassung aus der Rehabilitation einen Rentenantrag gestellt?

❑ Ja ❑ Nein

Falls Sie **„Ja“** angekreuzt haben, beantworten Sie bitte folgende Fragen:

- Wann haben Sie diesen Antrag gestellt (in welchem Monat)? _______________

- Was für eine Rente haben Sie beantragt:

❑ Erwerbsunfähigkeitsrente

❑ Altersrente

❑ Altersteilzeit

- Ist Ihr Rentenantrag schon bewilligt worden?

❑ Ja ❑ Nein

Falls Sie **„Ja“** angekreuzt haben, ab welchem Monat gilt dieser Rentenbescheid?

____

3. Fanden Sie das Ausfüllen dieses Fragebogens

- ☐ Sehr schwierig
- ☐ Schwierig
- ☐ Einfach
- ☐ Sehr einfach

4. Bitte geben Sie an, wie lange Sie ungefähr benötigt haben, um den Fragebogen auszufüllen.

5. Bitte tragen Sie hier Kommentare ein, die Sie gerne machen möchten und die uns helfen könnten, Ihre Antworten und aufgetretene Schwierigkeiten besser zu verstehen.

Vielen Dank für Ihre Hilfsbereitschaft
