# Supplementary material for: Development and first assessment of a questionnaire for health care utilization and costs for cardiac patients
Source: BMC Health Serv Res. 2008 Sep 19;8:187. doi: 10.1186/1472-6963-8-187 (PMC2556330; doi:10.1186/1472-6963-8-187)
Supplement: Additional file 2 — Cost questionnaire BMC English (Cost Diary). Straightforward translation of the original German version (additional file 1). See also disclaiming remarks before using it. [file 1472-6963-8-187-S2.pdf]

## Disclaimer

The following cost questionnaire is a straightforward translation of the original German version which was developed and assessed as described in the concomitant article.
The authors want to note that the translational process did not use forward and backward translation techniques as this was not the main goal of the article and beyond the scope of the study. The English version should therefore be taken as a useful starting point for a more in depth adaptation. This should also take then into count the peculiarities of the service provision in the country in which the questionnaire is to be used.

BS, HH, RL

# Cost Diary

*How much does your illness cost?*

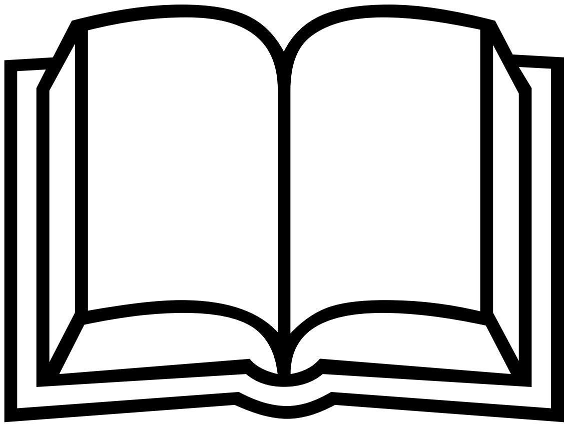

**Date:**_______________________________

**Name:** _______________________________

**Address:** _______________________________

_______________________________

Ulmer Rehaverbund: Pilotstudie kardiologische Rehabilitation
Klinik Schwabenland und Abteilung Gesundheitsökonomie, Universität Ulm

# Instructions for filling out the cost diary

Please fill out this diary three months after your discharge from the Clinic.

While filling out, please keep in mind that for this study, only expenditures and benefits related to your heart trouble are of importance. Should you, for example, have visited a doctor due influenza and would have been prescribed medication, please do not enter this into the cost book.

Please go over the following pages step-by-step, unhurriedly. Even if our cost diary may seem bulky to you on a first glance, you will not need much time for answering the questions. It is very unlikely, that you will be able to answer all questions.

Please, answer each question. If **„No“** applies, you may directly move on to the next question. If “Yes” applies, please also fill out the associated table.
There you may put several statements one below the other, e.g. if you took several types of drugs.

Sometimes you will be asked for multiple statements about an issue. For example, if you accepted help from relatives, please enter the accrued time. Should there have also been expenses, please enter that into the table as well.

*Example:*

| **Help**<br>(Kind of help) | Overall expenditure of time and costs per week |
|---|---|
| Help from relatives, friends or acquaintances | Hours: 4<br>€: 20,- |

In this way, please go through every question, one after the other. Keep in mind, that sometimes instead of number of contacts or costs, you will be asked for different statements.

On the last page, you will find several answers to questions which may arise while filling out the cost diary.

If you completed the cost diary, please put it into the enclosed post-paid envelope and send it back.

We assure you, that we will handle your data confidentially.

As a thank-you for your effort, for both completed and returned cost diaries, you will receive a **gift certificate valued at 12 €**.

**Please note:**

- Please only make statements which are related to your heart disease.
- Please return the cost diary in the enclosed post-paid envelope to the clinic.

**Dear study participant, please note:**

**The following questions apply to the period of**

# ***The last 4 Weeks***

| **4 weeks ago** | **3 weeks ago** | **2 weeks ago** | **Last week** |
|---|---|---|---|
| Mon | Mon | Mon | Mon |
| Tue | Tue | Tue | Tue |
| Wed | Wed | Wed | Wed |
| Thu | Thu | Thu | Thu |
| Fri | Fri | Fri | Fri |
| Sat | Sat | Sat | Sat |
| Sun | Sun | Sun | Sun |

**For a better illustration of the concerning period, we marked the last 4 weeks on the enclosed date sheet, starting with ______________.**

**1.** During the last 4 weeks, due to your heart disease, did you have to accept **help from relatives, friends or from professional services** for work, which you usually carry out by yourself? For example for housekeeping or for shopping

**No** ❑ **Yes** ❑

If you have checked "Yes" please complete the subsequent table 1. If you have checked "No" please move on to question number 2.

**Table 1**

Please insert here the average time in hours of help and the average cost of help per week. In case you do not know the cost please insert a question mark

| Kind of help | Average duration per week | Average cost per week |
|---|---|---|
| Help from relatives, friends or acquaintances | \|______\| hrs. | \|______\| € |
| Home help | \|______\| hrs. | \|______\| € |
| Professional aids (e.g.: Red cross) | \|______\| hrs. | \|______\| € |
| | \|______\| hrs. | \|______\| € |
| | \|______\| hrs. | \|______\| € |
| | \|______\| hrs. | \|______\| € |

2. During the last 4 weeks, due to your heart disease, did you use your **private car** or **public transportation** for rides, which you would not have done without having the disease (e.g. to go to see your doctor)

**No** ❑ **Yes** ❑

If you have checked **"Yes"** please complete the subsequent table 2. If you have checked **"No"** please move on to question number 3.

**Table 2**

Please enter the number of kilometres you drove by car, respectively the costs, that aroused from taking other means of transportation. If you do not know any of these, please enter a question mark.

| Means of transportation (kind) | Number of trips (outward and return voyage equates to one trip) | Kilometres or overall cost of trips |
|---|---|---|
| Car | \|______\| | \|______\| km |
| Tramway/Bus (cost of tickets) | \|______\| | \|______\| € |
| Train (cost of tickets) | \|______\| | \|______\| € |
| Taxi (fares) | \|______\| | \|______\| € |
| | \|______\| | \|______\| km/€ |
| | \|______\| | \|______\| km/€ |

**3.** <u>During the last 4 weeks</u>, have you regularly been active for prevention or relief of your heart disease, e.g. by going to ambulatory cardiac rehabilitation groups, riding the bike or going to the gym?

**No** ☐ **Yes** ☐

If you have checked **"Yes"** please complete the subsequent table 3. If you have checked **"No"** please move on to the second part of the questionnaire.

**Table 3**

Please enter the number of activities. In the third column, please enter your estimate if this number equals to the same amount or more activities, as compared to the last two months.

| Activities | Average number of activities per week | As compared to the last two months | | |
|---|---|---|---|---|
| | | less | same | more |
| Ambulatory cardiac rehabilitation groups | \|______\| | ☐ | ☐ | ☐ |
| Riding bike | \|______\| | ☐ | ☐ | ☐ |
| Gym | \|______\| | ☐ | ☐ | ☐ |
| Swimming | \|______\| | ☐ | ☐ | ☐ |
| | \|______\| | ☐ | ☐ | ☐ |
| | \|______\| | ☐ | ☐ | ☐ |
| | \|______\| | ☐ | ☐ | ☐ |

**Dear study participant, please note:**

**The following questions apply to the period of:**

# *The last 3 months*

| 3 months ago | 2 months ago | Last month |
|---|---|---|
| 1. | 1. | 1. |
| 2. | 2. | 2. |
| 3. | 3. | 3. |
| .. | .. | .. |
| .. | .. | .. |
| .. | .. | .. |
| 29. | 29. | 29. |
| 30. | 30. | 30. |
| 31. | | 31. |

**For a better illustration, we marked the concerning period of the last three months from _____________ until _____________ on the enclosed date sheet.**

**1.** During the last 3 months, have you seen a **physical therapist, alternative practitioner or other non-physician health care providers**? If so, which types of treatments did you make use of? (e.g. exercise therapy)

**No** ☐ **Yes** ☐

If you have checked **"Yes"** please complete the subsequent table 1. If you have checked **"No"** please move on to question number 2.

**Table 1**

| **Therapist (specialization)** | Treatments ( please give short description) | Number of treatments | Cost of all visits in € |
|---|---|---|---|
| Physical therapist | 1. ____________<br>2. ____________ | \|______\|<br>\|______\| | \|______\|<br>\|______\| |
| alternative practitioner | 1. ____________<br>2. ____________ | \|______\|<br>\|______\| | \|______\|<br>\|______\| |
| other non physician services | 1. ____________<br>2. ____________ | \|______\|<br>\|______\| | \|______\|<br>\|______\| |
| | 1. ____________<br>2. ____________ | \|______\|<br>\|______\| | \|______\|<br>\|______\| |
| | 1. ____________<br>2. ____________ | \|______\|<br>\|______\| | \|______\|<br>\|______\| |

1. During the last three months, due to your heart disease, have you had to see a doctor (e.g. your **general practitioner, cardiologist or psychologist**)(also telephone calls) or to make an appointment for a home visit? If „yes“, which types of treatments did you receive? (e.g. ECG, blood test, x-ray, ultrasound or filled prescription)

**No** ☐ **Yes** ☐

If you have checked **“Yes”** please complete the subsequent table 2. If you have checked **“No”** please move on to question number 3.

**Table 2**

| **GP and specialist visits** (specialization) | Number of contacts during the last 3 months | What was done? | How often? |
|---|---|---|---|
| General Practitioner | \|_____\| | ☐ ECG<br>☐ Blood test<br>☐ X-ray<br>☐ Ultrasound<br>☐ Prescription filled<br>☐ Telephone call<br>☐ ________________ | \|_____\|<br>\|_____\|<br>\|_____\|<br>\|_____\|<br>\|_____\|<br>\|_____\|<br>\|_____\| |
| Internist/ Cardiologist | \|_____\| | ☐ ECG<br>☐ Blood test<br>☐ X-ray<br>☐ Ultrasound<br>☐ Prescription filled<br>☐ Telephone call<br>☐ ________________ | \|_____\|<br>\|_____\|<br>\|_____\|<br>\|_____\|<br>\|_____\|<br>\|_____\|<br>\|_____\| |
| Psychologist | \|_____\| | ☐ Counselling<br>☐ ________________ | \|_____\|<br>\|_____\| |
| | \|_____\| | ☐ ________________ | \|_____\| |
| Ambulatory Care in hospital | \|_____\| | ☐ ________________ | \|_____\| |

**3.** During the last 3 months, have you seen a **doctor or therapist during your working hours**?

**No** ☐ **Yes** ☐

If you have checked **"Yes"** please complete the subsequent table 3. If you have checked **"No"** please move on to question number 4.

**Table 3**

| **Number of working hours, which you spent to see a doctor or therapist** | Overall loss of working time |
|---|---|
| Doctor visits | \|______\| hrs. |
| Therapist | \|______\| hrs. |
| | \|______\| hrs. |

**4.** During the last three months, did you attend **events/courses,** which were directly related to your disease or your heart-trouble e.g., information evenings, adult evening classes about low-fat nutrition or similar?

**No** ☐ **Yes** ☐

If you have checked **"Yes"** please complete the subsequent table 4. If you have checked **"No"** please move on to question number 5.

**Table 4**

| **Event/Course** | Cost / period |
|---|---|
| *Example: Autogenic training* | \|__40__\| €/ april – may 2002 |
| | \|______\| € ______________ |
| | \|______\| € ______________ |
| | \|______\| € ______________ |

**5.** During the last 3 months, have you been on sick leave due to your heart disease (at home or in hospital)?

**No** ☐ **Yes** ☐

If you have checked **"Yes"** please complete the subsequent table 5. If you have checked **"No"** please move on to question number 6.

**Table 5**

| **Time you were on sick leave** | Date from - until | Date from - until | Date from - until |
|---|---|---|---|
| Medically certified absence from work | from ___. ___. ___<br>until ___ . ___ .___ | from ___. ___. ___<br>until ___ . ___ .___ | from ___. ___. ___<br>until ___ . ___ .___ |
| Reduced hours of work for gradual reintegration | from ___. ___. ___<br>until ___ . ___ .___ | from ___. ___. ___<br>until ___ . ___ .___ | from ___. ___. ___<br>until ___ . ___ .___ |

**6.** <u>During the last three months</u>, due to your heart disease, were you in **hospital** or a **rehabilitation clinic**?

**No** ☐ **Yes** ☐

If you have checked **“Yes”** please complete the subsequent table 6. If you have checked **“No”** please move on to question number 7.

**Table 6**

| **Hospital/ Rehabilitation spells** | Date from - until | Date from – until | Date from - until |
|---|---|---|---|
| Hospital spells | from ___. ___.___ until ___ . ___ .___ | from ___. ___.___ until ___ . ___ .___ | from ___. ___.___ until ___ . ___ .___ |
| Reason for admittance | ________________ | ________________ | ________________ |
| Procedure | ☐ surgery<br>☐ other: ________________ | ☐ surgery<br>☐ other: ________________ | ☐ surgery<br>☐ other: ________________ |
| Rehabilitation stays | from ___. ___.___ until ___ . ___ .___ | from ___. ___.___ until ___ . ___ .___ | from ___. ___.___ until ___ . ___ .___ |

**1.** <u>During the last three months</u>, did you take drugs for your heart disease or for your risk factors (e.g. high blood pressure, cholesterol), which was **prescribed by a doctor** or did you buy **yourself pharmaceuticals**?

**No** ☐ **Yes** ☐

If you have checked „**Yes**“, please complete the subsequent tables 7a until 7c. If you have checked **„No“**, please move on to question 8.

**Table 7a**

Please enter the name of the **prescribed pharmaceutical**, which you **currently** take. Looking at the pharmaceutical, you can simply copy the information from the box. With pills, please specify the quantity and with drops, please specify the number of drops per day.

| **Prescribed pharmaceutical** (name) | Form of administration (tablest, coated pills, drops, ...) | Prescribed daily dosage |
|---|---|---|
| *Example: Aspirin 100N* | *Tablets* | *1-0-1* |
| | | |
| | | |
| | | |
| | | |
| | | |
| | | |
| | | |
| | | |
| | | |
| | | |

**7b** <u>During the last 3 months</u>, was there a **change** in your medication?

As a reminder, we made a list of your last medication on the enclosed sheet.

**No** ☐ **Yes** ☐

If you checked „**Yes**“, please describe the character of this change in the subsequent table:

**Table 7b**

| **Pharmaceutical** (name + Form of administration) | Change | Daily dosage | Date |
|---|---|---|---|
| *Example: Aspirin 100N pills* | ☒ newly prescribed<br>☐ discontinued<br>☐ dosage changed | *1 – 0 – 1* | since *10.05.02* |
| *or: Aspirin 100N pills* | ☐ newly prescribed<br>☐ discontinued<br>☒ dosage changed | *from 1 – 1 – 1 to 1 – 0 – 1* | since *10.05.02* |
| | ☐ newly prescribed<br>☐ discontinued<br>☐ dosage changed | | since ___ . ___ . ___ |
| | ☐ newly prescribed<br>☐ discontinued<br>☐ dosage changed | | since ___ . ___ . ___ |
| | ☐ newly prescribed<br>☐ discontinued<br>☐ dosage changed | | since ___ . ___ . ___ |
| | ☐ newly prescribed<br>☐ discontinued<br>☐ dosage changed | | since ___ . ___ . ___ |
| | ☐ newly prescribed<br>☐ discontinued<br>☐ dosage changed | | since ___ . ___ . ___ |
| | ☐ newly prescribed<br>☐ discontinued<br>☐ dosage changed | | since ___ . ___ . ___ |
| | ☐ newly prescribed<br>☐ discontinued<br>☐ dosage changed | | since ___ . ___ . ___ |

**Table 7c**

Please enter the pharmaceuticals, which you bought **yourself** due to your heart disease (without prescription). If you do not remember the costs per package, please enter a question mark.

| **Pharmaceutical without prescription** (name) | Number of packages | Costs per package | Package size (please check) | | |
|---|---|---|---|---|---|
| | | | N1 | N2 | N3 |
| | |_____| | |_____| € | | | |
| | |_____| | |_____| € | | | |
| | |_____| | |_____| € | | | |
| | |_____| | |_____| € | | | |
| | |_____| | |_____| € | | | |
| | |_____| | |_____| € | | | |

**8.** During the last 3 months, did you, e.g. in connection with your heart disease buy yourself **medical aids**, e.g. ergometer, books, blood pressure meter? Did you even have do modification measures (e.g. build in a stairlift)? Or did you have further expenditures?

**No** ☐ **Yes** ☐

If you have checked „Yes“, please fill out the subsequent tables 8a und 8b.

**Table 8a**

Should one of the examples in table 8a apply, please check it and enter the related price. With further medical aids, please enter name and amount of expenses.

| **Medical Aids** (examples) | Costs in € |
|---|---|
| ☐ Ergometer<br>☐ Pulse monitor<br>☐ Blood pressure meter | \|______\|<br>\|______\|<br>\|______\| |
| ☐ Books<br>☐ Cassettes<br>☐ Videos | \|______\|<br>\|______\|<br>\|______\| |
| ☐ Modification measure<br>☐ Other:<br>____________________________ | \|______\|<br>\|______\| |

**Table 8b**

In the subsequent table 8b, you may enter all expenses, which you had due to your disease, which have not been accounted for so far.

| **Further expenses, which have not been accounted for so far** (name) | Costs in € |
|---|---|
| | \|________\| |
| | \|________\| |
| | \|________\| |
| | \|________\| |

## Frequently asked questions

**I have had the flu and have therefore visited my doctor, who certified me unfit for work. Do I have to enter the drugs, the doctor visit, the time of sick leave and the time in which I was not able to carry out my housework?**
No, please only enter things, which are directly related to your heart disease. We do not want to include all treatments and expenses, you had due to other diseases.

**I cannot remember the exact information on the package of my drug. What should I enter?**
Please try to answer as exactly as possible. If you do not have the package of the drug for copying the name from it anymore, please enter the type of drug, e.g. beta blocker.

**I have not received the bill for my alternative practitioner visit yet. What should I do now?**
Please enter a question mark. Unless, from experience, you are quite sure how much the bill will be. In that case, please enter your estimate.

**Due to my heart disease, I have had expenses, which I do not know where to put, because none of the tables seems to really apply to them. Where should I enter these expenses?**
Please enter all expenses, treatments and applications which you cannot clearly assign to any question, into table 8b. Please do not leave out anything that is related to your heart disease.
